# The bovine dialyzable leukocyte extract, immunepotent CRP, synergically enhances cyclophosphamide-induced breast cancer cell death, through a caspase-independent mechanism

**DOI:** 10.17179/excli2022-5389

**Published:** 2023-01-13

**Authors:** Ana Luisa Rivera-Lazarín, Ana Carolina Martínez-Torres, Rafael de la Hoz-Camacho, Olga Liliana Guzmán-Aguillón, Moisés Armides Franco-Molinaa, Cristina Rodríguez-Padilla

**Affiliations:** 1 Universidad Autónoma de Nuevo León, Facultad de Ciencias Biológicas, Laboratorio de Inmunología y Virología, Monterrey 66455, Mexico; 2 LONGEVEDEN S.A. de C.V.

**Keywords:** cyclophosphamide, synergistic effect, breast cancer, cell death, chemotherapy, apoptosis

## Abstract

Breast cancer (BC) is one of the leading causes of cancer death worldwide. Cyclophosphamide (CTX) remains a mainstay in cancer therapy despite harmful adverse effects and cell death-resistances. To face this, combinational therapy of chemotherapies and immunotherapies has been proposed. IMMUNEPOTENT CRP (ICRP) is an immunotherapy that has cytotoxic effects in several cancer cells without affecting peripheral blood mononuclear cells (PBMC) and CD3+ cells. The aim of this study was to evaluate cytotoxicity, the type of cytotoxic effect, and several features involved in cell death induced by the combination of CTX with ICRP (ICRP+CTX) in breast cancer cells as well as their effect on healthy cells. For this purpose, human and murine breast cancer cells, MCF-7, MDA-MB-231 and 4T1, or PBMC were treated for 24 hours with ICRP, CTX or ICRP+CTX in different combination ratios for the assessment of cell death. Flow cytometry and microscopy were used to determine biochemical and morphological characteristics of cell death. Assays showed that ICRP in combination with CTX induce potentiated cell death manifested with morphological changes, loss of mitochondrial membrane potential, reactive oxygen species (ROS) production, and caspase activation. In addition, it was determined that ICRP+CTX-cell death is caspase-independent in all the breast cancer cells assessed. On the other hand, ICRP did not affect CTX-cytotoxicity in PBMC. For all the above, we can propose that the combination of ICRP with CTX an effective combination therapy, promoting their use even in tumoral cells with defects on proteins implicated in the apoptotic pathway.

## Introduction

Breast cancer (BC) is one of the leading causes of women death among cancers worldwide. Chemotherapy remains a mainstay in BC treatment and mostly in triple negative-breast cancer (TNBC) (ACS, 2019[1]), the most lethal BC subtype. Cyclophosphamide (CTX) is an alkylating agent that interferes with DNA replication and is one of the most widely used chemotherapies for BC (Emadi et al., 2009[8]). CTX is considered a first-line treatment against BC, despite its immunosuppressive effects (Rasmussen and Arvin, 1982[29]) when used in high-doses, and even if BC cells can develop cell death-resistance to CTX (Ji al., 2019[14]). 

One of the principal issues leading to high mortality in TNBC is chemoresistance, which is in great part caused by BC-cell death evasion. The principal cell death pathway induced by chemotherapies, including CTX, is apoptosis, a regulated cell death mechanism characterized by caspase-dependence. Since cancer cells evade cell death at multiple stages during tumorigenesis and metastasis, cells develop numerous ways to inhibit apoptosis, therefore, impairing the sensitivity of tumor cells to conventional chemotherapies (Hanahan and Weinberg, 2011[11]; Nedeljković and Damjanović, 2019[23]; Tait and Green, 2008[35]). To face this issue, drug combination therapy has been proposed as an interesting approach to overcome negative secondary effects associated with chemotherapy, and chemo-resistance (Apetoh et al., 2015[3]). 

Depending on the measurement of the drug combination effect, drug combinations could be defined as synergistic, additive, or antagonistic by several models that quantify the level of drug response. One of the most commonly used is the Chou-Talay combination index analysis, which allows the scoring of synergistic drug effects and avoiding ambiguities in the identification of effective combination treatments (Pemovska et al., 2018[27]; Chou and Talay, 1983[5]). Several studies testing drug combinations that include chemotherapies and immunotherapies have been carried out (Apetoh et al., 2015[3]) with several advantages such as improved efficacy over tumoral cells, while decreasing unwarranted toxicity over immune cells, decreased dosage at an equal or increased level of effectiveness, and counter chemo-resistance (Correia et al., 2018[6]). 

Recently, it has been described that an effective combinatorial regimen could be reached based on the targeting of different mechanisms of cell death (Correia et al., 2018[6]). In that sense, the bovine dialyzable leukocyte extract (bDLE), IMMUNEPOTENT CRP (ICRP), is an immunotherapy with cytotoxic potential in several cancer cell lines (Franco-Molina et al., 2006[10]) including breast cancer cells, without affecting non-cancerous cells (Martínez-Torres et al., 2020[22]; Lorenzo-Anota et al., 2020[19]). Furthermore, the combination of ICRP plus chemotherapy modifies the tumor microenvironment, potentiating and prolonging the antitumor effect (Santana-Krimskaya et al., 2020[32]). Additionally, ICRP alone or in combination with oxaliplatin (OXP) induced immunogenic cell death against murine melanoma (Rodríguez-Salazar et al., 2017[31]). ICRP is an immunogenic cell death inductor in breast cancer cells (Reyes-Ruiz et al., 2021[30]), and it has shown to improve the clinical parameters of breast cancer patients receiving standard chemotherapy schedules (Lara et al., 2010[17]). For all the above, this study aimed to investigate the effect and the mechanism of cell death of CTX in combination with ICRP in a panel of breast cancer cells including the most common subtype of BC, the luminal A, and the most aggressive BC-subtype, TNBC, likewise its effect in non-cancer cells.

## Methods

### Reagents 

IMMUNEPOTENT CRP^©^ (ICRP), a bovine dialyzable leukocyte extract, was produced by Laboratorio de Inmunología y Virología from Facultad de Ciencias Biológicas as previously described (Franco-Molina et al., 2006[10]). The product obtained from 1x10^8^ leukocytes is defined as one unit of ICRP. ICRP and Cyclophosphamide (Cryofaxol from Cryopharma; Tlajomulco de Zuñiga, Jalisco, México) were dissolved in complete DMEM-F12 or RPMI (GIBCO by Life Technologies, Grand Island, NY), as suitable. N-acetyl-L-cysteine (NAC) was dissolved in water. QVD.opH (QVD) was dissolved in dimethyl sulfoxide (DMSO). CTX, NAC and QVD (Sigma-Aldrich, St. Louis, MO) were wrapped in foil and stored following the manufacturer's instructions.

### Cell culture

Human breast adenocarcinoma MCF-7 (ATCC® HTB-22™), MDA-MB-231 (ATCC® HTB-26™), and murine breast adenocarcinoma 4T1 (ATCC® CRL-2539™) cells were obtained from the American Type Culture Collection (ATCC) and maintained at 37 °C in a humidified incubator containing 5 % CO_2_. MCF-7 and MDA-MB-231 cells were cultured in DMEM-F12 and 4T1 cells in RPMI-1640, both supplemented with 10 % fetal bovine serum (FBS) and 1 % penicillin-streptomycin (GIBCO) referred as complete DMEM or complete RPMI, respectively, and were routinely grown in 25-cm^3^ cell culture flasks (CORNING Enterprices, Corning, NY).

### Peripheral blood mononuclear cells (PBMC) isolation and death

Written informed consent was obtained from healthy donors from which a blood sample was obtained. PBMC isolation was made by density gradient centrifugation using Ficoll-Paque™ PLUS (GE Healthcare, Chicago, IL). The formation of cell layers was visualized, from which the population corresponding to PBMC was taken, maintained at 1x10^5^ cells per well in complete RPMI at 37 °C in 5 % CO_2_ atmosphere and treated using cytotoxic concentrations used in tumoral cells for 24 h, after which cell death was measured as explained in the following section.

### Cell death induction, pharmacological inhibition and analysis

For death induction, 5x10^4^ cells were treated with ICRP (0.5-1.45 U/mL) or CTX (5- 40 mM) to obtain the cytotoxic concentrations (CC) that were used to perform combination analysis. For the rest of the experiments, cells were exposed to ICRP, CTX and their combination (ICRP+CTX) in different combination-ratios for 24 h in 24-well dishes (Life Sciences). On the other hand, cells were co-treated with or without 30 min of 10 µM QVD or 5 mM NAC-pre-treatment for cell death inhibition assays. After treatment, cells were detached and washed twice with PBS and resuspended in 100 μL of binding buffer (10 mM HEPES/NaOH pH 7.4, 140 mM NaCl, 2.5 mM CaCl_2_) containing Annexin-V-APC (1 μg/mL, BD Pharmingen, San Jose, CA) and 0.5 μg/mL propidium iodide (PI, MilliporeSigma, Eugene, OR) staining to measure cell death with BD Accury6 flow cytometer (Becton Dickinson, Franklin Lakes, NJ) and analyzed using FlowJo Software (LCC, Ashland, OR).

### Morphological changes

Cell death associated-morphological changes were observed after 5x10^4^ cells were treated 24 h with ICRP, CTX, or their combination (ICRP+CTX) in the indicated concentrations, using an inverted microscope (Nikon Eclipse TS100) and bright-field-micrographs were taken with a Lummera INFINITY 1-2 CMOS 2.0 MP camera (20X). For this purpose, the focal position with the largest number of cells was selected in order to allow a better comparation of morphological changes. 

For chromatin condensation, cells (8x10^4^) were treated with ICRP, CTX, or their combination (ICRP+CTX) in the indicated concentrations for 24 h. Then, cells were washed with PBS and fixed using 4 % paraformaldehyde, after which cells were washed and 0.1 % triton was used for plasma membrane permeabilization. Hoechst staining (5 μg/mL) (SIGMA-ALDRICH) was added, then washed with PBS, and observed using a fluorescence microscope (OLYMPUS IX70) with objective 40X. Analysis was performed with Image-J software.

### ROS production analysis

ROS production-quantification was determined by staining cells with 2.5 μM HE (Hydroethidine) (Invitrogen, St. Louis, MO). Cells (5x10^4^) were treated and incubated in 24-well dishes (CORNING) with ICRP, CTX and their combination (ICRP+CTX) for 24 h. Cells were then harvested. The stain was in-cubated at 37 °C for 30 min, assessed by flow cytometry and analyzed as described above.

### Mitochondrial membrane potential analysis

To determine loss of mitochondrial membrane potential, 5x10^4^ cells in 24-well dishes (CORNING) were treated as mentioned before. Cells were then collected, stained using 20 nM tetramethyl rhodamine ethyl ester stain (TMRE, Sigma-Aldrich), incubated at 37 °C for 30 min, washed with PBS, and measured loss of TMRE-staining by flow cytometry as described above.

### Caspase activity assay

Cells (5x10^4^) in 24-well dishes (CORNING) were treated with ICRP, CTX and their combinations (ICRP+CTX) for 24 h. Cells were then harvested and stained following manufacturer's instructions. Caspase activity was determined through Generic Caspase Activity staining kit (TF2-VAD-FMK, Abcam, Cambridge, UK) using flow cytometry as described above.

### Statistical analysis

Results are presented as graphs that represent the mean ± SD of triplicate determinations from at least three independent experiments. Data were analyzed by GraphPad Prism (San Diego, CA), using paired Student's t-tests for *in vitro* studies, and unpaired Student's t-tests for cytotoxicity in PBMC studies, considering statistical significance as p<0.05.

## Results

### ICRP and CTX induce cell death as single agents through caspase-independent and caspase-dependent mechanisms, respectively, in breast cancer cells

**Figure 1:**
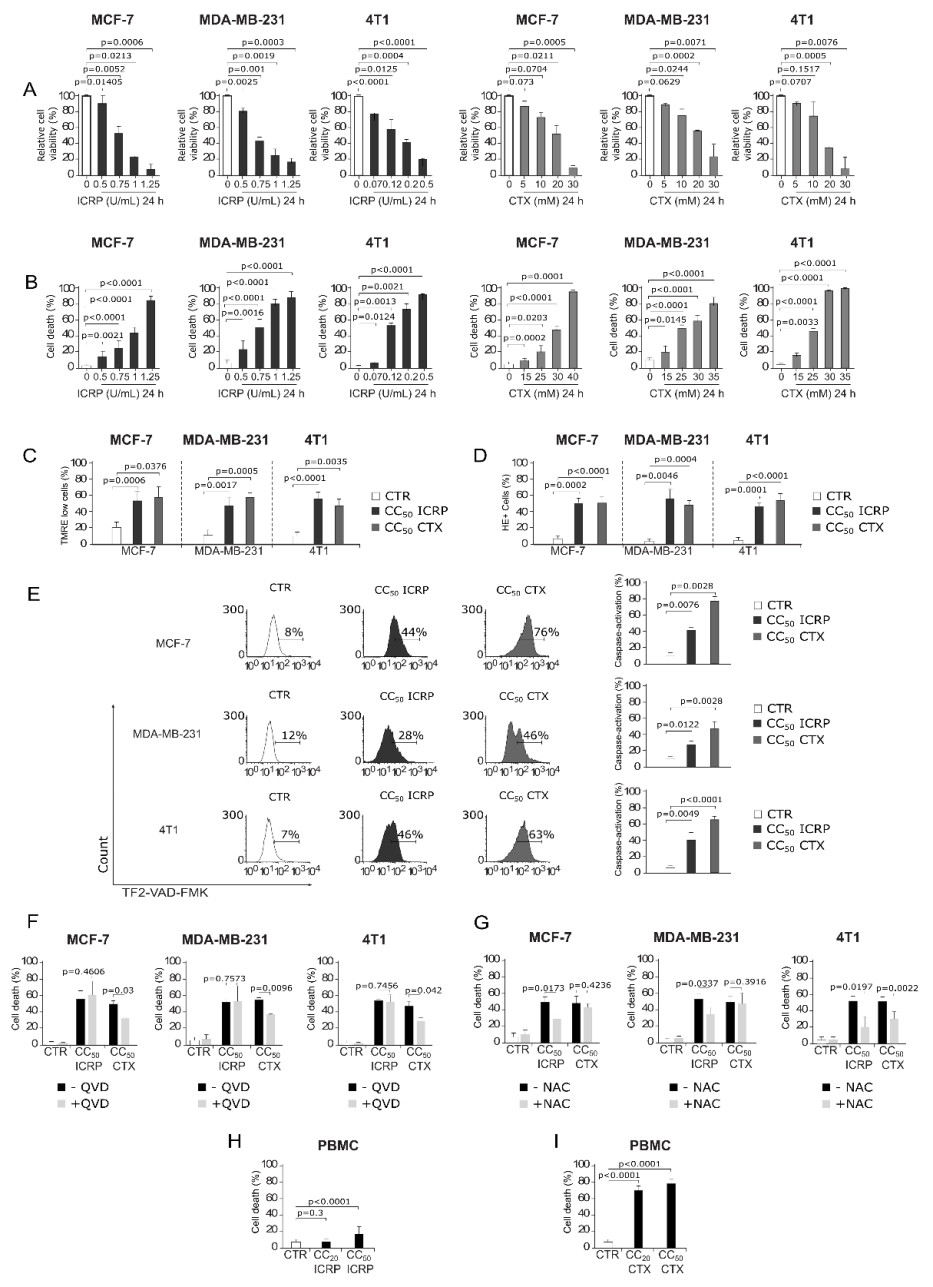
ICRP and CTX mechanisms of cell death in breast cancer cells and its effect in PBMC. MCF-7, MDA-MB-231 and 4T1 cells were treated for 24 h and biochemical features of cell death were evaluated and expressed as percentage. A) Relative cell viability was determined using MTT assay considering control cell's absorbance as 100 %. Flow cytometry was used to measure. B) Cell death analyzed by AnnV/PI staining. For the next evaluations, 1, 0.75 and 0.12 U/mL of ICRP were used as CC_50_ in MCF-7, MDA-MB-231 and 4T1 cells, respectively, as well as 30 mM for MCF-7, and 25 mM for MDA-MB-231 and 4T1 cells of CTX CC_50_-treatment. C) Loss of mitochondrial membrane potential determined using TMRE staining. D) ROS production analyzed by HE staining. E) Caspase activation measured by TF2-VAD-FMK. (F, G) Cell death obtained by AnnV/PI of cells pre-treated in presence or absence of F) QVD or G) NAC. (H, I) Cell death obtained by AnnV/PI of PBMC treated with H) ICRP or I) CTX. Graphs represent means ±SD of triplicates from at least three independent experiments.

MCF-7, MDA-MB-231 and 4T1 cell viability was determined after treatment with ICRP (dark gray) or CTX (gray). Results showed that ICRP and CTX diminish breast cancer cell viability as treatment concentration increases (Figure 1A(Fig. 1)). This cytotoxicity was correlated with cell death assays by AnnV/PI staining (Supplementary Figure 1). ICRP and CTX induced cell death in a concentration-dependent manner in the three cell lines tested (Fig. 1B(Fig. 1)). It was required 0.7, 0.5 and 0.09 U/mL of ICRP and 25 mM CTX for MCF-7 and 15 mM CTX for MDA-MB-231 and 4T1 cells to induce cell death in 20 % of cell population (CC_20_). On the other hand, cell death of 50 % of the cells (CC_50_) was reached at 1, 0.75 and 0.12 U/mL of ICRP in MCF-7, MDA-MB-231 and 4T1 cells, respectively, whereas CTX CC_50_ was 30 mM for MCF-7, and 25 mM for MDA-MB-231 and 4T1 cells. 

Furthermore, ICRP and CTX induced loss of mitochondrial membrane potential in 45-60 % in the three BC-cell lines tested after treatment with CC_50_ of each treatment for 24 h, as shown in Figure 1C(Fig. 1). Both, the CC_50_ of ICRP and the CC_50_ CTX, induced ROS production in around 45-55 % MCF-7, MDA-MB-231 and 4T1 cells (Figure 1D(Fig. 1)). Moreover, it was observed that while the CC_50_ of ICRP induced 28-42 % of cells with caspase activation, the CC_50_ CTX induced higher values, ranging from 48-77 % of caspase activation in the three-breast cancer cells lines assessed (Figure 1E(Fig. 1)). 

Despite ICRP and CTX induce similar characteristics in their cell death mechanism, no significant effect in cell death was observed with pre-treatment using the pan-caspase inhibitor, QVD, after treatment with ICRP. In contrast, QVD inhibited CTX-cell death significantly (Figure 1F(Fig. 1) and Supplementary Figure 2). On the other hand, the antioxidant NAC, significantly diminished ICRP-cell death in the cell lines tested, whereas only 4T1-death showed inhibition after CTX treatment using NAC (Figure 1G(Fig. 1) and Supplementary Figure 3). 

Furthermore, we analyzed cell death induced by ICRP and CTX at the higher concentrations used in breast cancer on PBMC. Figure 1H(Fig. 1) shows that ICRP only induces a low cytotoxicity in PBMC from healthy donors at CC_50_ of MCF-7 cells (1.0 U/mL), contrary to CTX which since CC_20 _of MCF-7 cells (25 mM), induces high cytotoxicity against PBMC (Figure 1I(Fig. 1) and Supplementary Figure 4).

All these differences in the mechanism of cell death induced by ICRP and CTX allowed us to hypothesize that a potentiated effect on cell death could be achieved by the combination of both treatments, caspase-dependent and -independent mechanisms.

### ICRP in combination with CTX synergistically potentiates the cytotoxicity of individual treatments against breast cancer cells but not PBMC

**Figure 2:**
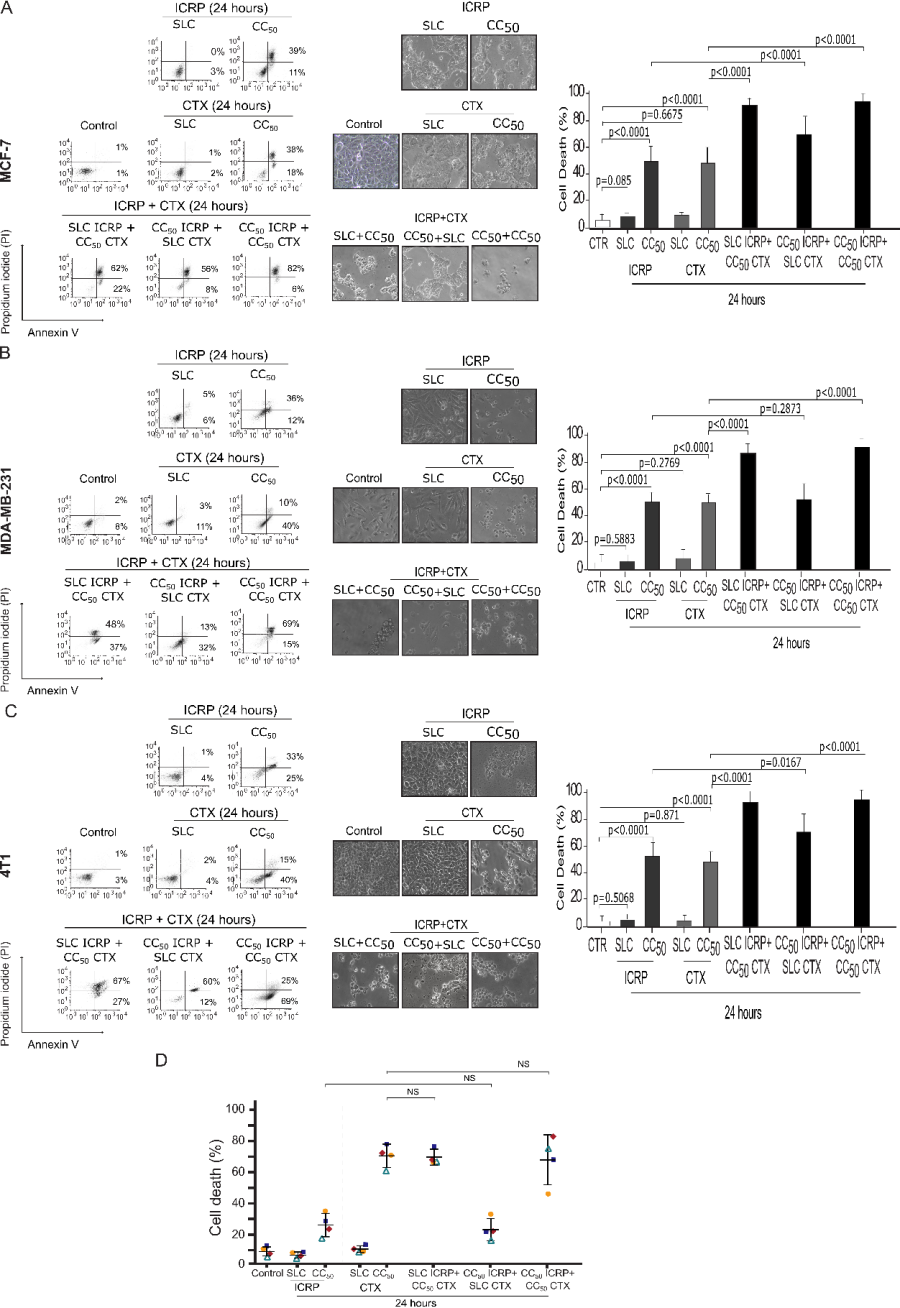
ICRP+CTX cytotoxicity in breast cancer cells and PBMC. MCF-7, MDA-MB-231, 4T1 cells and PBMC were treated for 24 h with ICRP, CTX and their combination at different ratios and analyzed by flow cytometry. AnnV/PI staining was used to analyze cell death of A) MCF-7, B) MDA-MB-231 and C) 4T1 cells. Representative dot plots are shown on the left, representative images of morphological changes were taken in bright field using an inverted microscope (20X), and graphs are on the right. D) Cell death of PBMC from healthy donors (n=4). Mean value of at least three independent experiments performed in triplicate ± SD were graphed.

Aiming for substantially diminishing the concentration of chemotherapy, different combination ratios were designed. First, we used a sublethal concentration (non-cytotoxic) of ICRP (SLC), in combination with CC_50_ CTX corresponding to each BC cell line. Furthermore, to investigate if CTX has an effect in the ICRP cell death, we tested a combination of CC_50_ ICRP with SLC CTX. Moreover, to examine the effect of equipotent concentrations of both treatments, we tested the combination of CC_50_ of ICRP and CTX. Results showed non-significant cell death induced by SLC of ICRP or CTX compared to non-treated MCF-7 (Figure 2A(Fig. 2)), MDA-MB-231 (Figure 2B(Fig. 2)) and 4T1 cells (Figure 2C(Fig. 2)). As left panel shows in Figure 2(Fig. 2), CC_50 _ICRP induces an increase in double-positive (Annexin V and PI) cell population in the three BC-cell lines, while CC_50 _CTX induces an increase mainly in Annexin V-positive dot plot and in double-positive cell population. When combining treatments in different combination ratios, analysis showed an increase predominantly in double-positive cell populations of MCF-7, MDA-MB-231 and 4T1. This combination ratios also induced morphological changes, demonstrating cell death-morphology like CTX's in MCF-7 treated cells. Moreover, right panel shows a significant increase in cell death of MCF-7 by SLC ICRP+CC_50_ CTX, CC_50_ ICRP+SLC CTX and CC_50_ ICRP+ CC_50_ CTX compared to single agents, reaching 91.9 %, 70.1 %, and 94.2 % of cell death (Figure 2A(Fig. 2)). In addition, morphological assessment showed cell confluence reduction and alterations in cell morphology involving rounding-up of the cell and retraction of the stellate projections of MDA-MB-231-treated cells. Analysis showed that SLC ICRP+CC_50 _CTX, and CC_50_ ICRP+CC_50_ CTX reached 86.9 % and 91.2 % of cell death, respectively, whereas CC_50_ ICRP+SLC CTX showed non-significant cell death compared to ICRP alone (52.6 %, Figure 2B(Fig. 2)). Likewise, 4T1 showed rounding-up of the cell, similar to the treatment with CTX alone, and cell death analysis showed a significant cell death increase to 93.2 %, 70.7 %, and 97 % cell death, when treated with SLC ICRP+CC_50 _CTX, CC_50_ ICRP+SLC CTX, and CC_50_ ICRP+CC_50_ CTX, respectively (Figure 2C(Fig. 2)). Additionally, we chose the highest cytotoxic concentration used in BC cells (MCF-7 ones) to assess the cytotoxic effect of ICRP+CTX in human peripheral blood mononuclear cells (PBMC). As Figure 2D(Fig. 2) shows, ICRP is not toxic in PBMC, as only a low cytotoxicity was observed at CC_50_ ICRP of MCF-7 (1.0 U/mL, 17.7 %). In addition, non-significant cell death was observed when PBMC were treated with SLC CTX. In contrast, CC_50_ CTX of MCF-7 (30 mM) induced high cell death in PBMC (78.7 %). Moreover, any of the combination ratios tested increased cell death, compared to its corresponding monotherapy, nor, compared to CTX alone in PBMC.

**Figure 3:**
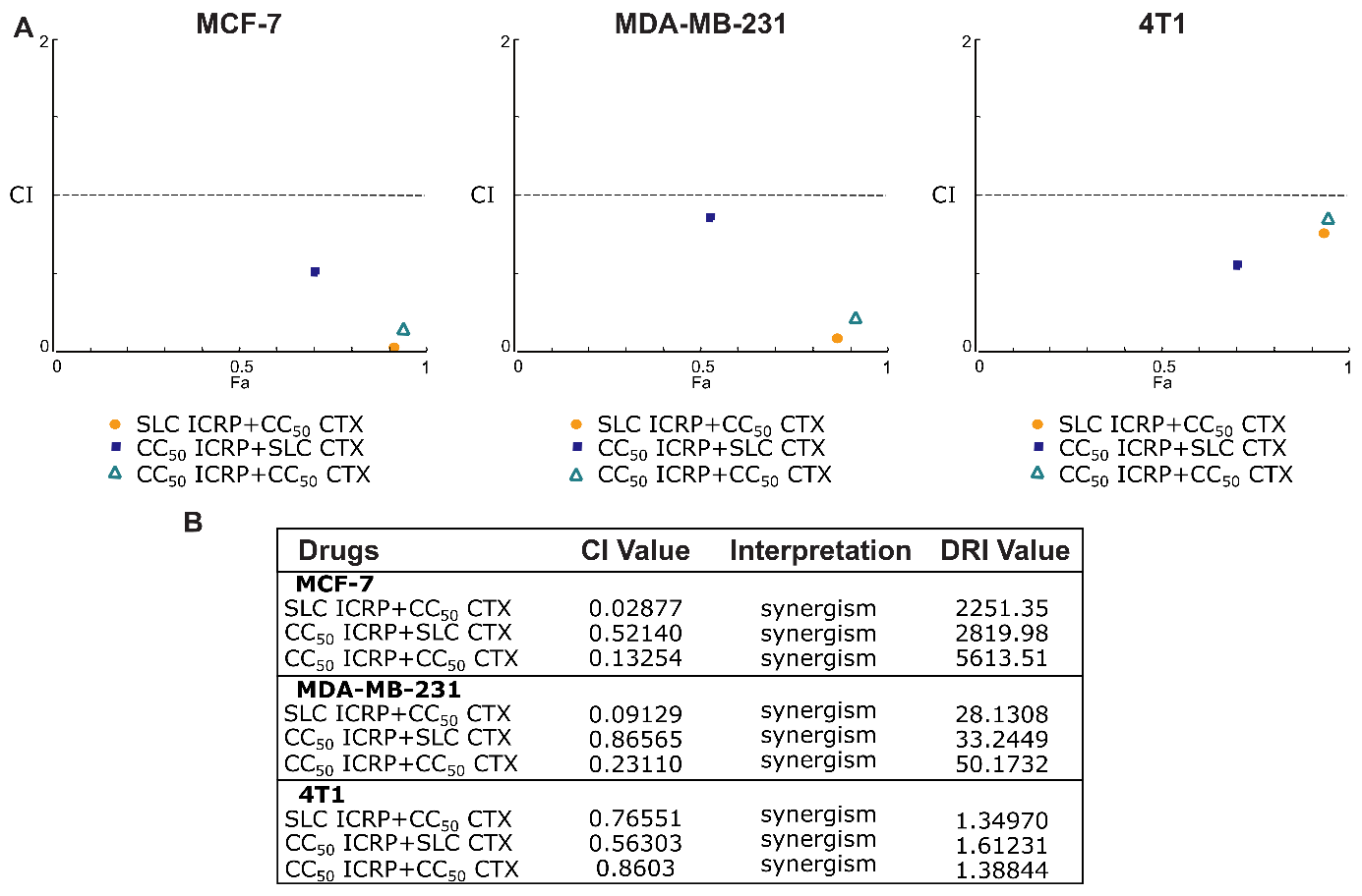
ICRP+CTX induces different cytotoxic effects in breast cancer cells. MCF-7, MDA-MB-231 and 4T1 were treated with the combination of ICRP+CTX in different ratios for 24 h and cell death was analyzed using the software Compusyn. A) Fa-CI graphs from cell death. B) table with the effect of combined ICRP and CTX treatment. CI<1 indicates synergism, additive effect is CI=1, and CI>1 represents antagonism.

Additionally, the drug interaction effect of ICRP and CTX was assessed by combination index (CI) determination. Figure 3A(Fig. 3) shows the Fa-CI graphs with the CI values that reveled synergistic effect (CI<1) being specific for the cancer cell line tested with shared synergistic effect at the combination of SLC ICRP+CC_50_ CTX and CC_50 _ICRP+CC_50_ CTX in the three cell lines assessed. Furthermore, to estimate the extent to which the dose of CTX can be reduced in combination to achieve a cytotoxic effect comparable to monotherapy, drug reduction index (DRI) was calculated. All the cell lines tested showed DRI values above 1. Figure 3B(Fig. 3) summarizes the values of CI and DRI.

For the shared synergistic effect in the three cell lines, SLC ICRP+CC_50_ CTX and CC_50_ ICRP+CC_50_ CTX were chosen to determine the main biochemical characteristics of ICRP+CTX cell death, evaluating features evoked by the monotherapies.

### ICRP in combination with CTX induce morphologic and mitochondrial alterations during cell death in breast cancer cells 

**Figure 4:**
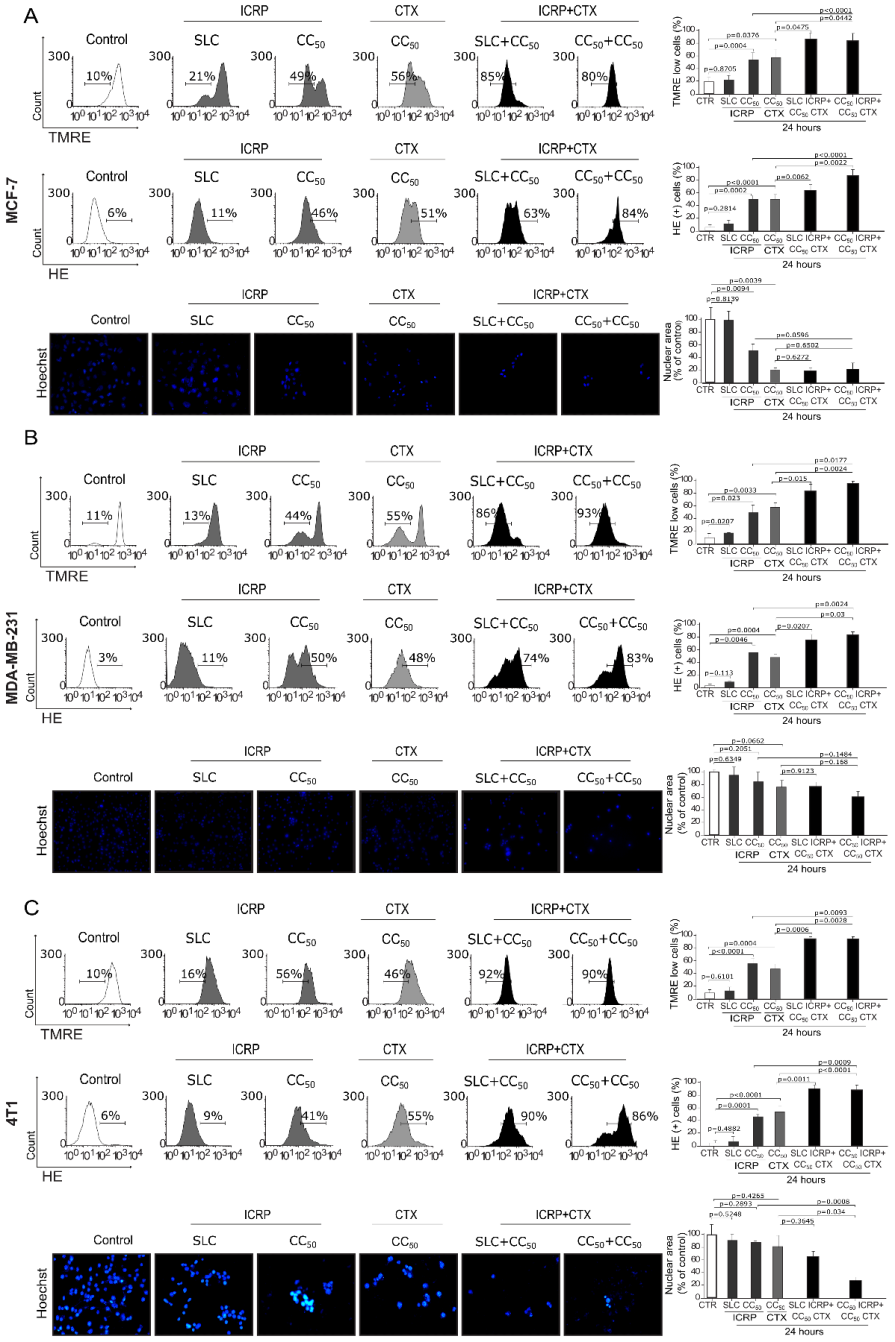
ICRP+CTX cell death causes morphologic and mitochondrial alterations in breast cancer cells. Cells were treated with the combination of ICRP+CTX in two different ratios for 24 h and analyzed by microscopy or flow cytometry. Representative histograms and graphs from loss of mitochondrial membrane potential and ROS production determined using TMRE and HE staining, respectively. Representative images of chromatin condensation analyzed using Hoechst staining by fluorescence microscopy in A) MCF-7, B) MDA-MB-231 and C) 4T1 cells. The mean value of at least three independent experiments performed in triplicate ± SD were graphed.

A significant augmentation in loss of mitochondrial membrane potential (87 % and 84.33 %) and ROS production (64.4 % and 86.9 %) was induced after treatment with SLC ICRP+CC_50_ CTX and CC_50_ ICRP+CC_50 _CTX in MCF-7 compared to single treatments. MCF-7-treated cells also showed nuclear condensation compared to control cells (p=0.0056 and p=0.0087, respectively) (Figure 4A(Fig. 4)). In MDA-MB-231, SLC ICRP+CC_50_ CTX and CC_50_ ICRP+CC_50 _CTX caused a significant increase in mitochondrial alterations demonstrated by 83.5 % and 98.98 %, respectively, of loss of mitochondrial membrane potential and 75.94 % and 83.4 %, respectively, of ROS production. Morphological assessment showed nuclear condensation induced by both ratios compared to control cells (p=0.0463 and p=0.0322, respectively) (Figure 4B(Fig. 4)). The 4T1-treated cells revealed 95.23 % of cells with loss of mitochondrial membrane potential and 90.46 % of cells with increased ROS production after treatment with SLC ICRP+CC_50 _CTX. Additionally, a significant increase of loss of mitochondrial membrane potential (95.28 %) and ROS production (88.41 %) was observed after CC_50_ ICRP+CC_50_ CTX-treatment, compared to single treatments. Moreover, both combination ratios showed significant nuclear condensation compared to control cells (p=0.026 and p=0.0154) (Figure 4C(Fig. 4)).

### ICRP in combination with CTX turns cell death in a caspase-independent mechanism in breast cancer cells

**Figure 5:**
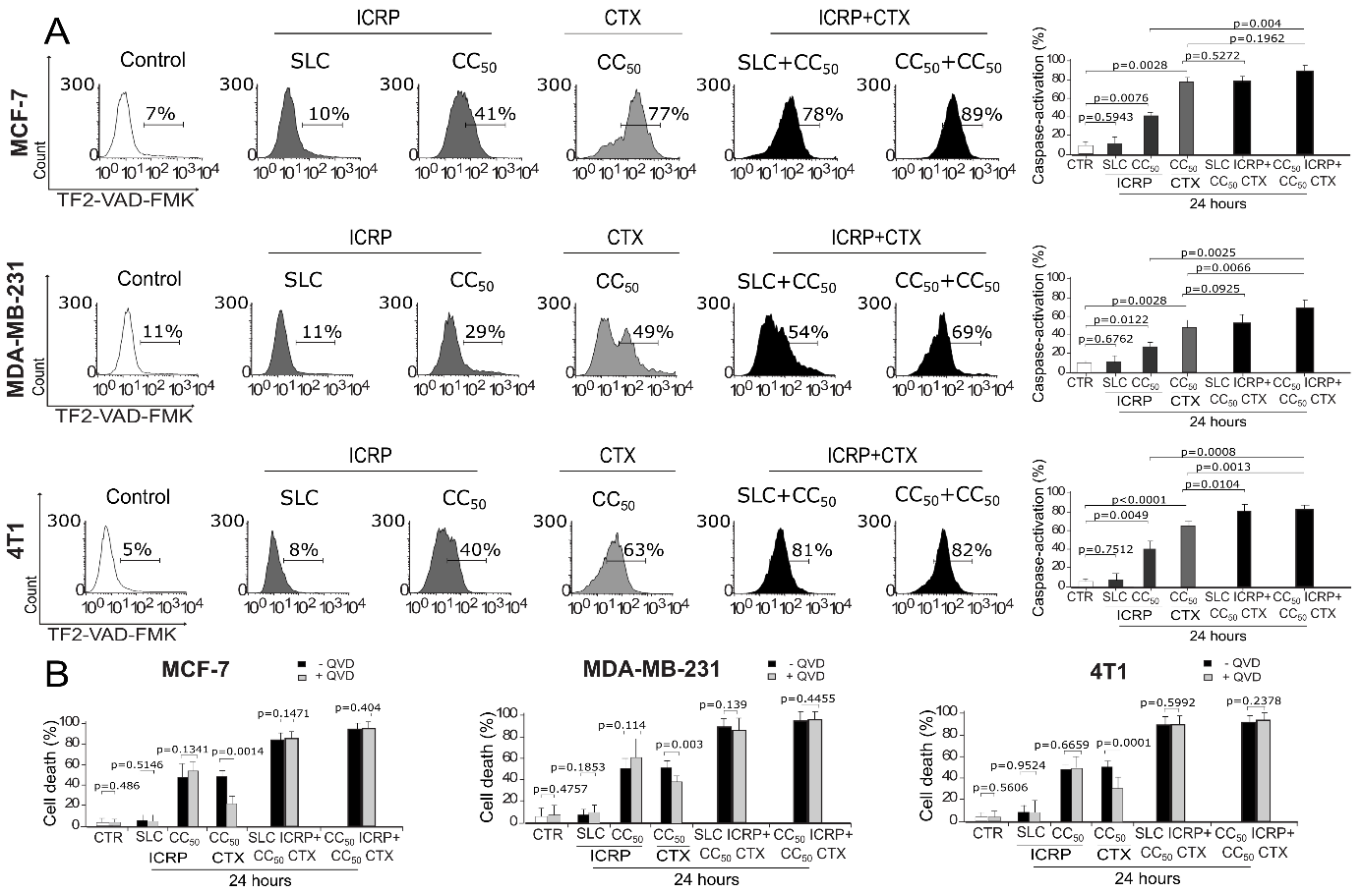
ICRP+CTX treatment induces cell death through a caspase-independent mechanism in breast cancer cells. Cells were treated with the combination of ICRP+CTX in two different ratios for 24 h and analyzed by flow cytometry in MCF-7, MDA-MB-231 and 4T1. A) Representative histograms and graphs obtained from caspase activation analysis determined by TF2-VAD-FMK staining. B) Cell death analyzed pre-treating MCF-7, MDA-MB-231 and 4T1, in presence or absence of caspase inhibitor (QVD, shown in light gray) using AnnV/PI staining. Graphs represent the mean value of at least three independent experiments performed in triplicate ± SD.

After evaluation of different features involved in ICRP+CTX cytotoxicity, we investigated if caspase activation takes place during cell death induced by these combinations. As Figure 5(Fig. 5) shows, SLC ICRP did not induce significant caspase activation, only at CC_50_ ICRP induced 28-42 %, however, CTX induced higher caspase activation ranging from 48-77 %. When we tested the combination of SLC ICRP+CC_50_ CTX, analysis showed no significant increase of caspase activation (78.89 % and 53.86 %) in MCF-7 and MDA-MB-231, respectively (Figure 5A(Fig. 5)), whereas 4T1 reached 81.3 % caspase activation, significantly higher compared to CTX-treated cells. Furthermore, although MCF-7 reached 89.75 % of caspase activation, a significant increase of caspase activation was observed in MDA-MB-231 and 4T1 cells treated with CC_50 _ICRP+CC_50 _CTX (69.37 % and 83.22 %, respectively) in comparison with CTX monotherapy (Figure 5A(Fig. 5)). Thus, ICRP+CTX induced caspase activation at equal or increased levels than CTX as monotherapy. 

As caspases were activated in combinations of ICRP plus CTX, we wondered if this type of cell death was caspase-dependent as CTX-treatment alone. Thus, we pharmacologically inhibited caspase activation using the pan-caspase inhibitor QVD. This inhibition resulted in non-significant differences in SLC ICRP+CC_50_ CTX and CC_50_ ICRP+CC_50_ CTX-mediated cell death in MCF-7, MDA-MB-231 and 4T1 (Figure 5B(Fig. 5)).

## Discussion

Cyclophosphamide is a mainstay chemotherapy for breast cancer; its mechanism of cell death has been explored in several studies, which has been associated with ROS production and caspase-dependent cell death (Emadi and Brodsky et al.,2009[8]; de la Hoz-Camacho et al., 2022[7]). On the other hand, Immunepotent CRP is a promising immunotherapy which modulates immune cells and induces non-apoptotic regulated cell death (caspase-independent) triggered by ROS production and involving mitochondrial and nuclear alterations in cells from cervical, lung and breast cancer cells, suggesting a conserved mechanism of cell death in solid tumors (Lorenzo-Anota et al., 2022[18]; Martínez-Torres et al., 2018[21] and 2019[20]; Reyes-Ruiz et al., 2021[30]); however, the cytotoxic effect of ICRP in combination with chemotherapies, including CTX, against breast cancer cells was reported here for the first time. 

To test the potential of ICRP to synergize CTX-cell death in breast cancer cells, we selected three BC cell lines with several molecular differences, thus, different response to treatment. MCF-7 is a model of luminal A subtype, whereas MDA-MB-231 and 4T1 cells are TNBC subtype. Our results showed that MDA-MB-231 and 4T1 cells are more sensitive to CTX than MCF-7 cells by its CC_50_ (25 mM for MDA-MB-231 and 4T1, and 30 mM for MCF-7). The response to chemotherapy was previously compared between luminal A and TNBC, indicating that even though luminal A is less sensitive to chemotherapy than TNBC, this last subtype has high probability of developing chemotherapy resistance (Harbeck et al., 2019[12]; Parker et al., 2009[26]). 

Throughout this work, we demonstrated that ICRP synergically potentiates the cell death induced by CTX in breast cancer cells, while sparing PBMC from healthy donors. Combinations of CC_50_ CTX with other treatments such as sclareol (Scl) and resveratrol (RES) used at SLC in breast cancer cells, reached 60-70 % inhibition of cell viability (Afshari et al., 2020[2]; Singh et al., 2009[33]). Remarkably, these results are different to the ones presented in this study, owing to when ICRP was used at SLC in combination with CC_50_ CTX, our results showed 86-93 % cell death in all the cell lines tested here, demonstrating the potential of ICRP in potentiating CTX-cell death, since low doses. The use of very low doses of the monotherapies in combination is an interesting approach since one of the drugs is inactive individually, but active in combination, reaching favorable outcomes, such as enhanced efficacy with decreased dosage, as we demonstrated by CI and DRI values.

On the other hand, previously it has been reported combinations of treatments at high doses (CC_50_) such as thymoquinone (TQ) and RES, with low doses of CTX (SLC) tested in breast cancer cells. CTX at low dose significantly augmented the inhibition in cell viability induced by TQ, reaching values of 82-100 % inhibition of viability, while CTX in combination with RES resulted in modest cytotoxic activity (22 %) (Khan et al., 2019[16]; Singh et al., 2009[33]). In this study, CTX had variable cytotoxic effects depending on the cell line tested, ranging from 52-70 % cell death. All these results together suggest that the capacity of CTX to augment cell death, depends on the cytotoxic compound which is combined with. Nevertheless, SLC CTX maintained or increased the cell death induced by CC_50_ ICRP, depending on the cell line tested, this result reflected in the statistical analysis as synergistic or additive effect. These results could be explained by the fact that quantification of CI is based in an equation considering the biological effect of each drug according to its dose-effect curve and the fraction affected by the combination of the treatments (Chou, 2006[4]). 

The cytotoxicity induced by combinations using equipotent concentrations (CC_50_) of CTX with Scl, RES and Naringenin (Nar), ranged 75-93 % (Afshari et al., 2020[2]; Singh et al., 2009[33]; Noori et al., 2020[24]). Comparably, our results showed that CC_50_ ICRP+CC_50_ CTX induced 91-97 % cell death, supporting the potential of ICRP to amplify the effect of CTX.

Noteworthy, none of the studies described above are based on rigorous drug combination methods since its responses were only evaluated by statistical analysis but have not been experimentally scored as we did here. It has been suggested that drug-combination analysis using a rigorous method must be done in order to avoid errors in assessing synergism (Jia et al., 2009[15]). Our results highlight the relevance of specifically defining the level of drug synergy by quantification methods and point out that ICRP is more efficacious in combinational therapy against breast cancer cells when used at similar or lower doses than CTX.

Additionally, to test the effect of the combination therapy on immune system cells, with human peripheral blood mononuclear cells, we demonstrated that ICRP does not augment the cytotoxicity induced by CTX, which is desirable for a combination of treatments in which one of those, is known to have high cytotoxic effects in immune system cells, such as the induced by CTX and epirrubicin (EPI), another chemotherapy for the treatment of breast cancer, reported by de la Hoz-Camacho et al. (2022[7]). Using HEK 293 (human embrionic kidney cells) and MDCK cells (Medin-Darby canine kidney), Singh et al., showed modest cytotoxicity induced by the combination of CTX with RES ranging from 5-15 % inhibition of cell viability. However, these values were higher than the induced by CTX as monotherapy in these cells (7 %) (Singh et al., 2009[33]).

Moreover, specific mechanism of cell death has only been fully elucidated for a few of the explored drug combinations. Knowledge of the molecular mechanisms induced by combination therapy can provide clues that favor the discovery and optimization of new successful drug combinations based on a rational design (Jia et al., 2009[15]; Pemovska et al, 2018[27]). Previous studies have reported drug combinations with synergistic effects, most of them involving drugs with the same pathway of action, whose mechanism of cell death is preserved by the combination of those agents (Tanaka et al., 2005[36]; Pennati et al., 2005[28]; Humeniuk et al., 2007[13]).

Experimental evidence has demonstrated shared characteristics of the mechanism of cell death such as caspase activation induced by the combination of CTX with sclareol in breast cancer cells, likewise the caspase activation and caspase-mediated enhanced cell death induced by the combination of CTX with resveratrol (Afshari et al., 2020[2]; Singh et al., 2009[33]). Yet, we found no reports where the pathway induced by the combination of an agent that induces caspase-independent and a second agent that induces caspase-dependent cell death, switches in the mechanism of cell death activated. Most of the studies of combinations involving CTX, have demonstrated caspase-mediated enhanced cell death, for instance, the combination of resveratrol (RES) with CTX induces caspase-dependent cytotoxic activity on MCF-7 cells (Singh et al., 2009[33]; Pang et al., 2011[25]). Other drug-combinations induce a caspase-independent cell death mechanism in breast cancer cells, such as Mn porphyrin in combination with ascorbate which mediates caspase-independent breast cancer cell death (Evans et al., 2014[9]). Here we found that inhibition of caspases resulted in non-significant changes in cell death induced by ICRP+CTX, suggesting that ICRP+CTX-regulated cell death is different from apoptosis. Remarkably, this finding in the caspase-independent cell death induced by ICRP+CTX occurs even when ICRP is in the lowest dose (SLC ICRP) of the combination ratio, leading us to future studies to elucidate the exact molecular mechanisms involved in this transition. Furthermore, this brings us to hypothesize that the mechanism of cell death induced by the combination of ICRP with CTX may represent an advantage in resistant-tumoral cells with defects on proteins implicated in the apoptotic pathway (Suparji et al., 2016[34]). However, there are still many questions and additional perspectives regarding the effector of the ICRP+CTX-mediated cell death.

**Figure 6:**
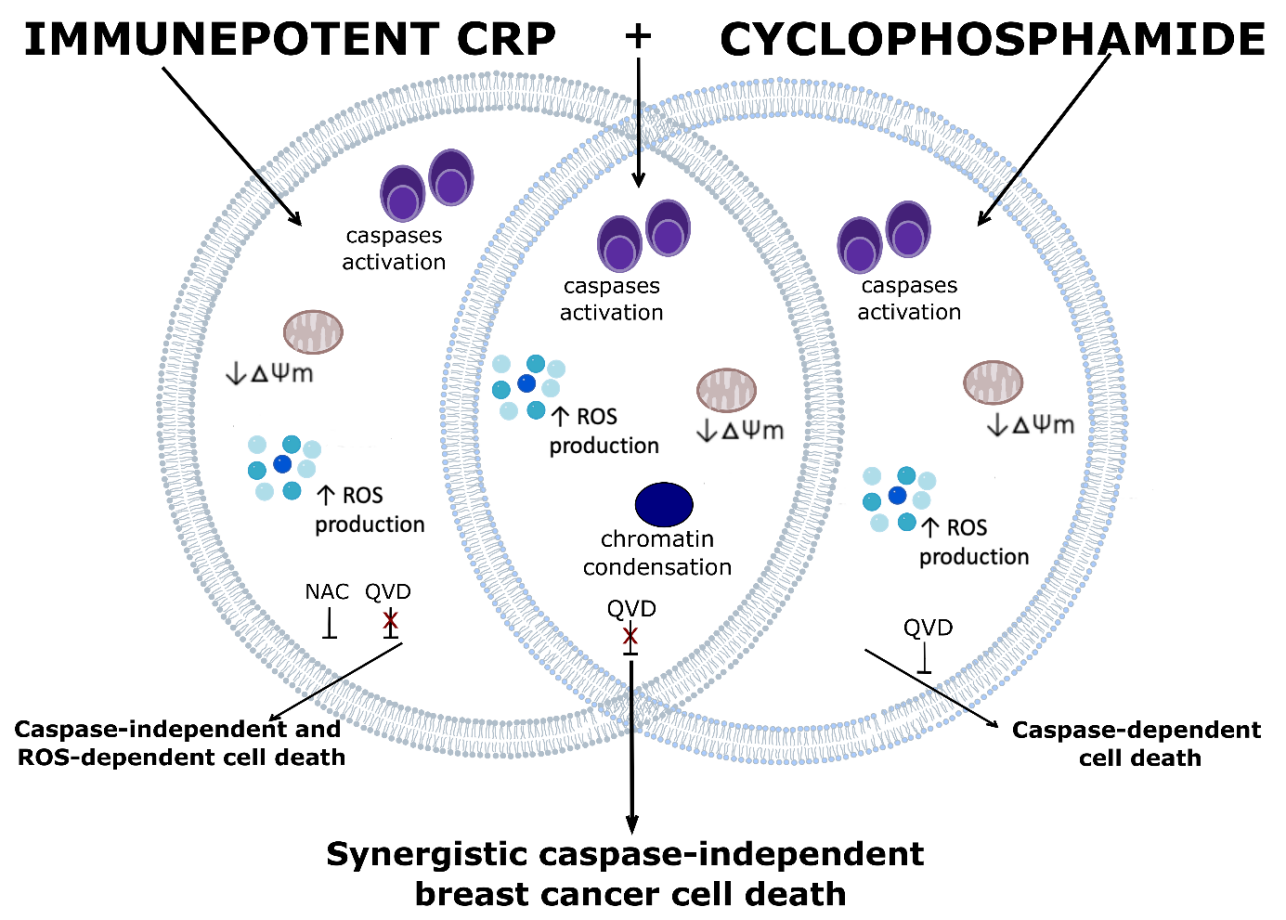
ICRP+CTX cell death depiction in breast cancer cells. ICRP induces caspase-independent and ROS-dependent breast cancer cell death, whereas CTX induces caspase-dependent breast cancer cell death. Here we showed that ICRP in combination with CTX induces synergistic cell death manifested with morphological changes and it involves mitochondrial alterations such as the loss of mitochondrial membrane potential and reactive oxygen species (ROS) production, and even though caspase activation occurs, ICRP+CTX cell death is caspase-independent in all the breast cancer cells assessed.

In conclusion, the combination of Immunepotent CRP with cyclophosphamide triggers synergistic cell death, involving loss of mitochondrial membrane potential, an increase in ROS production, caspase activation, morphological changes, and caspase-independent cell death in breast cancer cells, while ICRP did not affect CTX-cytotoxicity in PBMC, allowing to reduce the CTX doses. Overall, our results show that the combination of ICRP and CTX may be used to overcome treatment resistance (Figure 6(Fig. 6)).

## Notes

Ana Luisa Rivera-Lazarín and Ana Carolina Martínez-Torresa contributed equally as first author.

## Declaration

### Acknowledgments

ALRL, RHC and OLGA thank CONACYT for the scholarship provided.

### Conflict of interest

The authors have no conflict of interest to declare.

## Supplementary Material

Supplementary information
