# Supplementary material for: The bovine dialyzable leukocyte extract, immunepotent CRP, synergically enhances cyclophosphamide-induced breast cancer cell death, through a caspase-independent mechanism
Source: EXCLI J. 2023 Jan 13;22:131–45. doi: 10.17179/excli2022-5389 (PMC10043454; doi:10.17179/excli2022-5389)
Supplement: Supplementary information [file EXCLI-22-131-s-001.pdf]

EXCLI Journal 2023;22:131-145 – ISSN 1611-2156
Received: September 11, 2022, accepted: January 09, 2023, published: January 13, 2023

Supplementary information to:

Original article:

THE BOVINE DIALYZABLE LEUKOCYTE EXTRACT, IMMUNEPOTENT CRP, SYNERGICALLY ENHANCES CYCLOPHOSPHAMIDE-INDUCED BREAST CANCER CELL DEATH, THROUGH A CASPASE-INDEPENDENT MECHANISM

Ana Luisa Rivera-Lazarín [a,+], Ana Carolina Martínez-Torres[a,+]*, Rafael de la Hoz-Camacho[a], Olga Liliana Guzmán-Aguillón[a], Moisés Armides Franco-Molina[a], Cristina Rodríguez-Padilla[a,b]

[a] Universidad Autónoma de Nuevo León, Facultad de Ciencias Biológicas, Laboratorio de Inmunología y Virología, Monterrey 66455, México
[b] LONGEVEDEN S.A. de C.V.

[+] Co-first authors
* **Corresponding author:** Ana Carolina Martínez-Torres, Universidad Autónoma de Nuevo León, Facultad de Ciencias Biológicas, Laboratorio de Inmunología y Virología, Monterrey 66455, México. E-mail: ana.martinezto@uanl.edu.mx

https://dx.doi.org/10.17179/excli2022-5389

This is an Open Access article distributed under the terms of the Creative Commons Attribution License (http://creativecommons.org/licenses/by/4.0/).

EXCLI Journal 2023;22:131-145 – ISSN 1611-2156
Received: September 11, 2022, accepted: January 09, 2023, published: January 13, 2023

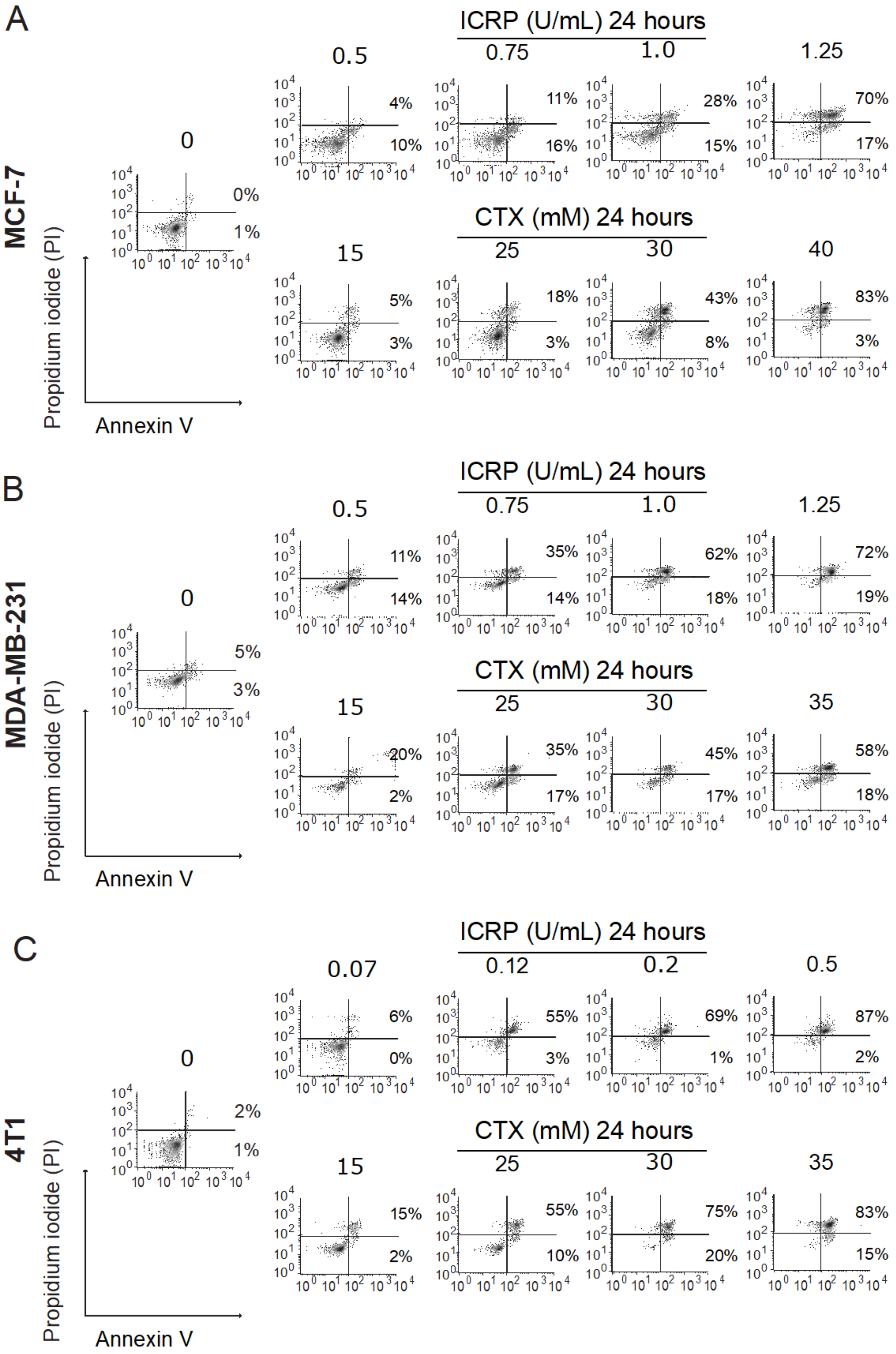

Supplementary Figure 1: A) MCF-7, **B**) MDA-MB-231 and **C**) 4T1 cells were treated for 24 h. Flow cytometry was used to measure cell death analyzed by AnnV/PI staining. Representative dot plots from at least three independent experiments.

EXCLI Journal 2023;22:131-145 – ISSN 1611-2156
Received: September 11, 2022, accepted: January 09, 2023, published: January 13, 2023

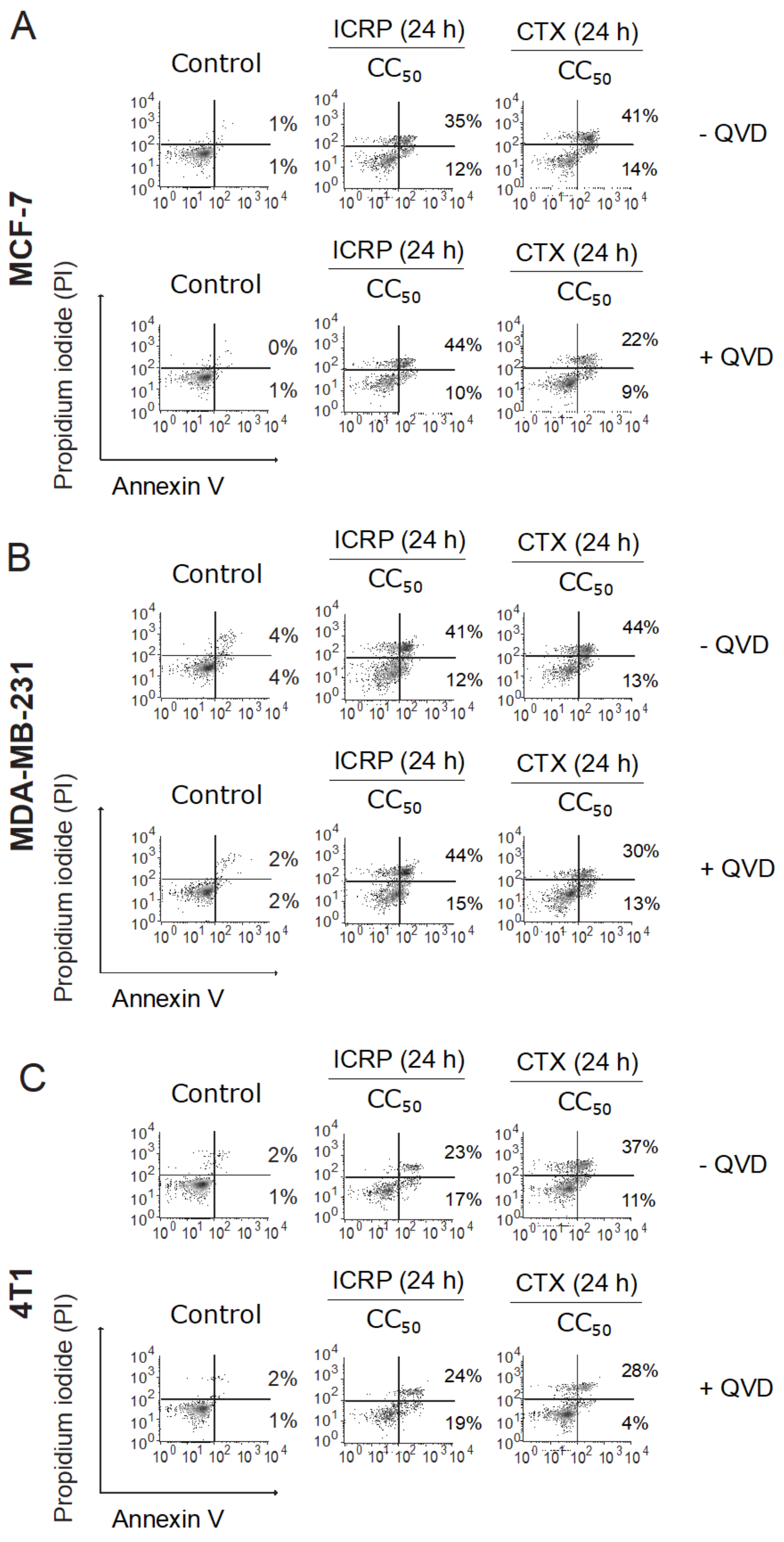

Supplementary Figure 2: Representative dot plots of cell death obtained by AnnV/PI of **A**) MCF-7, **B**) MDA-MB-231 and **C**) 4T1 cells pre-treated in presence or absence of QVD, and then treated with ICRP CC_{50} or CTX CC_{50} for 24 h, using flow cytometry.

EXCLI Journal 2023;22:131-145 – ISSN 1611-2156
Received: September 11, 2022, accepted: January 09, 2023, published: January 13, 2023

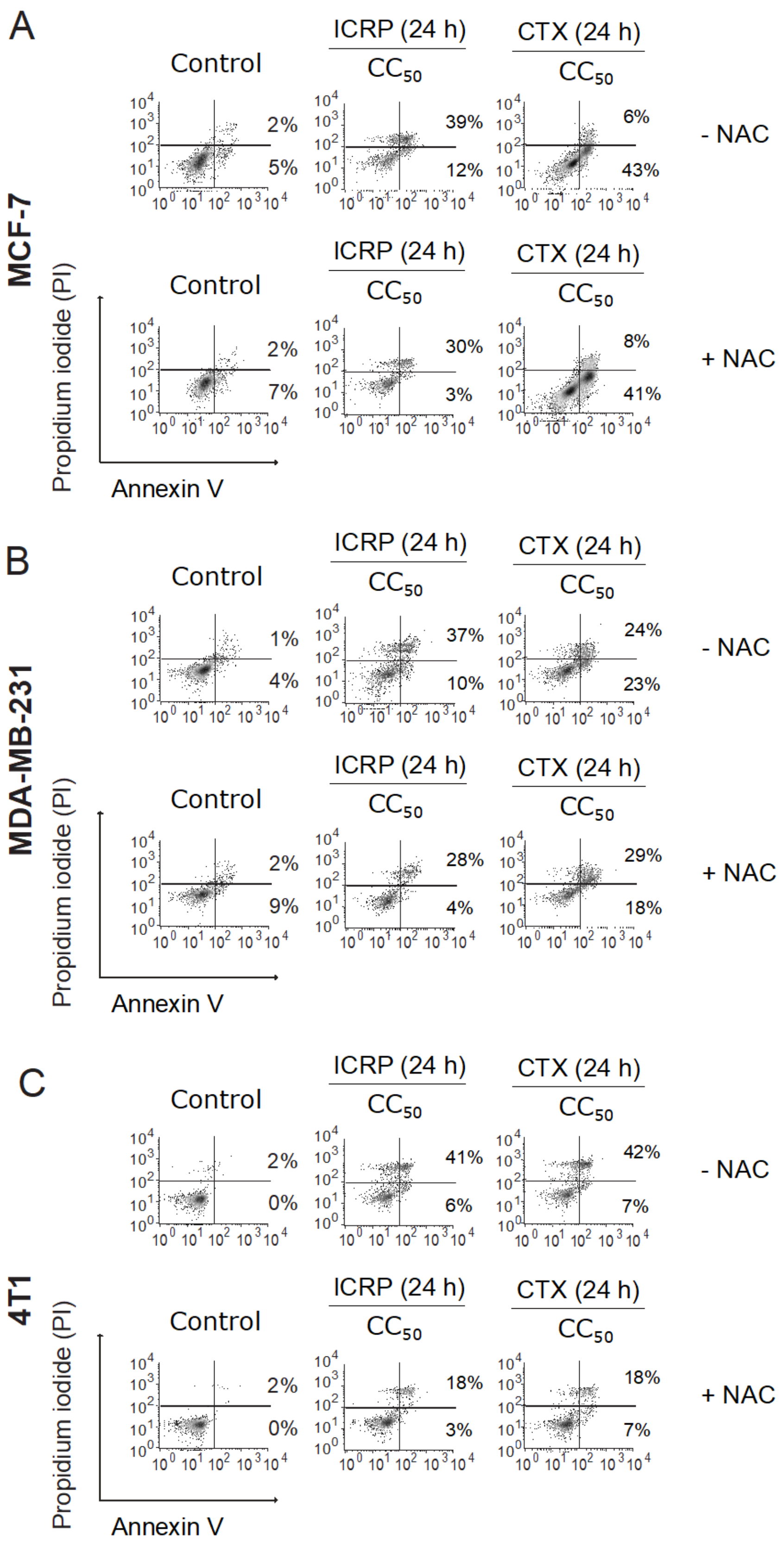

Supplementary Figure 3: Representative dot plots of cell death obtained by AnnV/PI of **A**) MCF-7, **B**) MDA-MB-231 and **C**) 4T1 cells pre-treated in presence or absence of NAC, and then treated with ICRP CC_{50} or CTX CC_{50} for 24 h, using flow cytometry.

EXCLI Journal 2023;22:131-145 – ISSN 1611-2156
Received: September 11, 2022, accepted: January 09, 2023, published: January 13, 2023

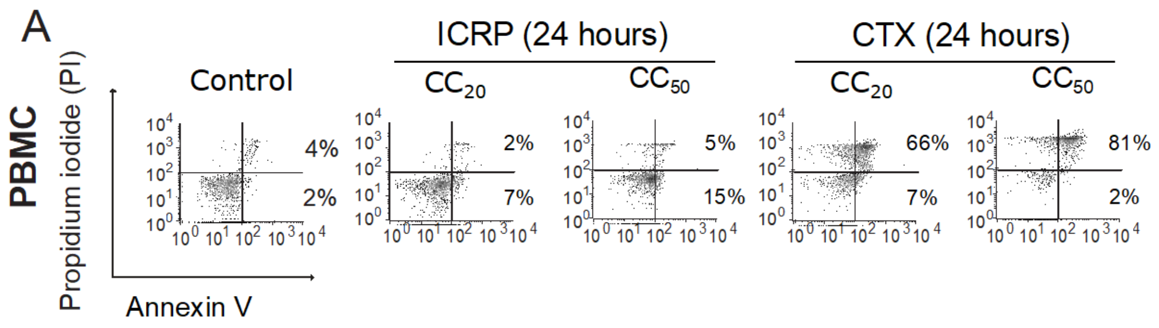

Supplementary Figure 4: PBMC obtained from healthy donors were treated with CC_{20} or CC_{50} of ICRP or CTX for 24 h and cell death was evaluated by AnnV/PI staining using flow cytometry. **A**) Dot plots are representative of means of triplicates from at least three independent experiments.
